# Framing the Human-Centered Artificial Intelligence Concepts and Methods: Scoping Review

**DOI:** 10.2196/67350

**Published:** 2025-05-28

**Authors:** Roberta Bevilacqua, Tania Bailoni, Elvira Maranesi, Giulio Amabili, Federico Barbarossa, Marta Ponzano, Michele Virgolesi, Teresa Rea, Maddalena Illario, Enrico Maria Piras, Matteo Lenge, Elisa Barbi, Garifallia Sakellariou

**Affiliations:** 1 Scientific Direction, IRCCS INRCA, Via Santa Margherita 5, Ancona, Italy, 39 0718004767; 2 Intelligent Digital Agents (IDA) Research Group, Fondazione Bruno Kessler (FBK), Trento, Italy; 3 Department of Health Sciences, Section of Biostatistics, University of Genoa, Genoa, Italy; 4 Department of Public Health, University of Naples "Federico II", Naples, Italy; 5 Department of Endocrinology, Diabetes, Andrology and Nutrition, Federico II University Hospital, Naples, Italy; 6 Digital Health & Wellbeing, Fondazione Bruno Kessler, Trento, Italy; 7 Neuroscience and Human Genetics Department, Meyer Children's Hospital IRCCS, Florence, Italy; 8 Department of Internal Medicine and Therapeutics, University of Pavia, Pavia, Italy; 9 General Medicine, Istituti Clinici Scientifici Maugeri SpA SB, IRCCS, Pavia, Italy

**Keywords:** human-centered artificial intelligence, HCAI, usability, acceptability, design principles, UCD, human-centered design, AI principles, ethics, AI applications, machine learning, ML, algorithm, analytics, predictive model, predictive analytics, deep learning, early warning, early detection, review, user-centered design

## Abstract

**Background:**

With the rapid expansion of artificial intelligence (AI) applications, researchers have begun focusing on the concept of human-centered artificial intelligence (HCAI). This field is dedicated to designing AI systems that augment and improve human abilities, rather than substituting them.

**Objective:**

The objective of the paper was to review the information on design principles, techniques, applications, methods, and outcomes adopted in the field of HCAI, in order to provide some insights on the discipline, in relation with the broader concepts of human-centered and user-centered design.

**Methods:**

Following the Preferred Reporting Items for Systematic Reviews and Meta-Analyses Extension for Scoping Reviews (PRISMA-ScR) checklist guidelines, we conducted a scoping review in PubMed, ScienceDirect, and IEEE Xplore, including all study types, excluding narrative reviews and editorials.

**Results:**

Out of the 1035 studies retrieved, 14 studies conducted between 2018 and 2023 met the inclusion criteria. The main fields of application were the health sector and AI applications. Human-centered design methodologies were adopted in 3 studies, personas in 2 studies, while the remaining methodologies were adopted in individual studies.

**Conclusions:**

HCAI emphasizes designing AI systems that prioritize human needs, satisfaction, and trustworthiness, but current principles and guidelines are often vague and difficult to implement. The review highlights the importance of involving users early in the development process to enhance trust, especially in fields like health care, but notes that there is a lack of standardized HCAI methodologies and limited practical applications adhering to these principles.

## Introduction

User-centered design (UCD) is an iterative methodology that places the user at the center of the design of innovative solutions, allowing the information gathered since its early stages to define the product features and end user experience. This approach is typically enabled by interdisciplinary teams and different methodologies that work together synergistically to optimize the user experience of systems, products, and processes.

The interaction of users with innovation prototypes not only accelerates the identification of usability issues, highlighting improvement opportunities, but also strengthens the capacity of researchers to define cost and benefits evaluation methods that are propaedeutic to identify the return of investments also in terms of economic benefit. The requirements and recommendations for human-centered design principles have been formalized in ISO 9241‐210, which details the role of UCD and its benefits for human-centered design applied to interactive technologies.

The use of appropriate UCD methods, especially when tailored to specific stakeholders’ contexts, can reduce the risk of a given product not meeting stakeholder requirements or being rejected by users.

Similar to UCD, human-centered design (HCD) is an approach that prioritizes human needs, capabilities, and behavior. Therefore, HCD aims to address problems by putting people with their human perspective at the center of the processes, involving them in all stages of problem solving, from observation to brainstorming, conceptualizing, solution development, and final implementation [1]. It is believed that such an approach can improve the usability of an innovation, increasing product acceptance and user satisfaction, proving effective in all situations where solutions are needed that are not only useful, but also involve the emotional sphere of users in some way [2-4].

Recently, due to the increasing growth of artificial intelligence (AI) applications, researchers have shifted their attention to the construct called human-centered artificial intelligence (HCAI), a discipline aimed at creating AI systems to amplify and enhance human capabilities, rather than replace them [5]. As HCD, HCAI places people at the center by seeking to improve, experience after experience, their lives [6]. On the basis of the design phase of any technological product, there are a number of useful techniques that can be adopted to obtain quick feedback from the end users, even in the absence of a fully functioning solution, to provide creative design alternatives to fit the users’ preferences and needs. If properly trained, HCAI could offer useful solutions tailored to the peculiar characteristics of the final users, for example, considering new approaches coming from the original combination of many variables.

Despite the undeniable richness of HCAI, there is the need to map the main concepts and constructs behind it, as well as to understand the methods and design principles that underpin it, in order to ensure that these metrics are adequately included in the experimental methodology and understand their role in supporting the UCD and HCD approach. The objective of this paper is to review the information on design principles, techniques, applications, methods, and outcomes adopted in the field of HCAI, in order to provide some insights on the discipline, in relation to the broader concepts of HCD and UCD.

## Methods

The scoping review was carried out according to the Preferred Reporting Items for Systematic Reviews and Meta-Analyses Extension for Scoping Reviews (PRISMA-ScR; see Checklist 1) [7]. Before the start of the project, a protocol was developed to guide the review process, shared among the authors and registered on the Open Science Framework (OSF) platform. The topic of interest for the review was any application of HCAI for UCD, in any population of adults. The outcomes of interest were descriptions of the HCAI approach, description of the HCAI design, outcomes of the application of HCAI, user involvement in HCAI, description of use cases, methods for understanding and the user’s mental model, description of user needs, and new working practices. Any other design methodology could serve as a comparator. All English-language studies, with the exception of narrative and editorial reviews, could be included. Clinical questions, exploring the applications of HCAI for design, were translated into epidemiological terms using the Patient, Intervention, Comparator, Outcome, and Study Type (PICOS) methodology (see Textbox 1).

Based on the identified PICOS, search strategies were created and applied to the electronic PubMed, ScienceDirect and IEEE Xplore databases from January 1, 2018, to October 27, 2023. Searches were performed by a single reviewer. The complete search strategies are presented in Multimedia Appendix 1.

The records retrieved from the search were imported into the review management platform Rayyan, where duplicate entries were removed. A total of 4 investigators (RB, EM, TB, and ML) working in pairs performed screening, selection, and data extraction. Specifically, 2 blinded reviewers assessed the title and abstracts to define eligibility for full-text assessment, and, at the subsequent step, assessed full-texts for inclusion. Disagreement was resolved by consensus. Data extraction from the included studies was performed by using a standardized extraction form, including general information on the paper, the field of application, design methods, the adopted model, design principles and properties, technology, and type of AI; extraction was performed by a reviewer and checked by a second one. Results were summarized in summary of findings tables. The methodological quality of the included studies was assessed by a single author (GS) with the SANRA (Scale for the Assessment of Narrative Review Articles) or the Critical Appraisal Skills Programme (CASP) checklist for qualitative studies, depending on the study design.

Textbox 1. **PICOS (Patient, Intervention, Comparator, Outcome, Study Type) driving the search strategies and the inclusion criteria.**

Patient:

- Any type of patient.

Intervention:

- Human-centered artificial intelligence (HCAI) for design.

Comparator:

- Any other technique adopted for design.

Outcome:

- Description of HCAI approaches.
- Description of HCAI design.
- Outcome of HCAI application.
- User involvement in HCAI.
- Description of study cases.
- Description of methods for understanding.
- Description of user’s mental model.
- User’s needs and new working practices.

Study type:

- All study types excluding narrative reviews and editorials.

## Results

**Figure 1.**
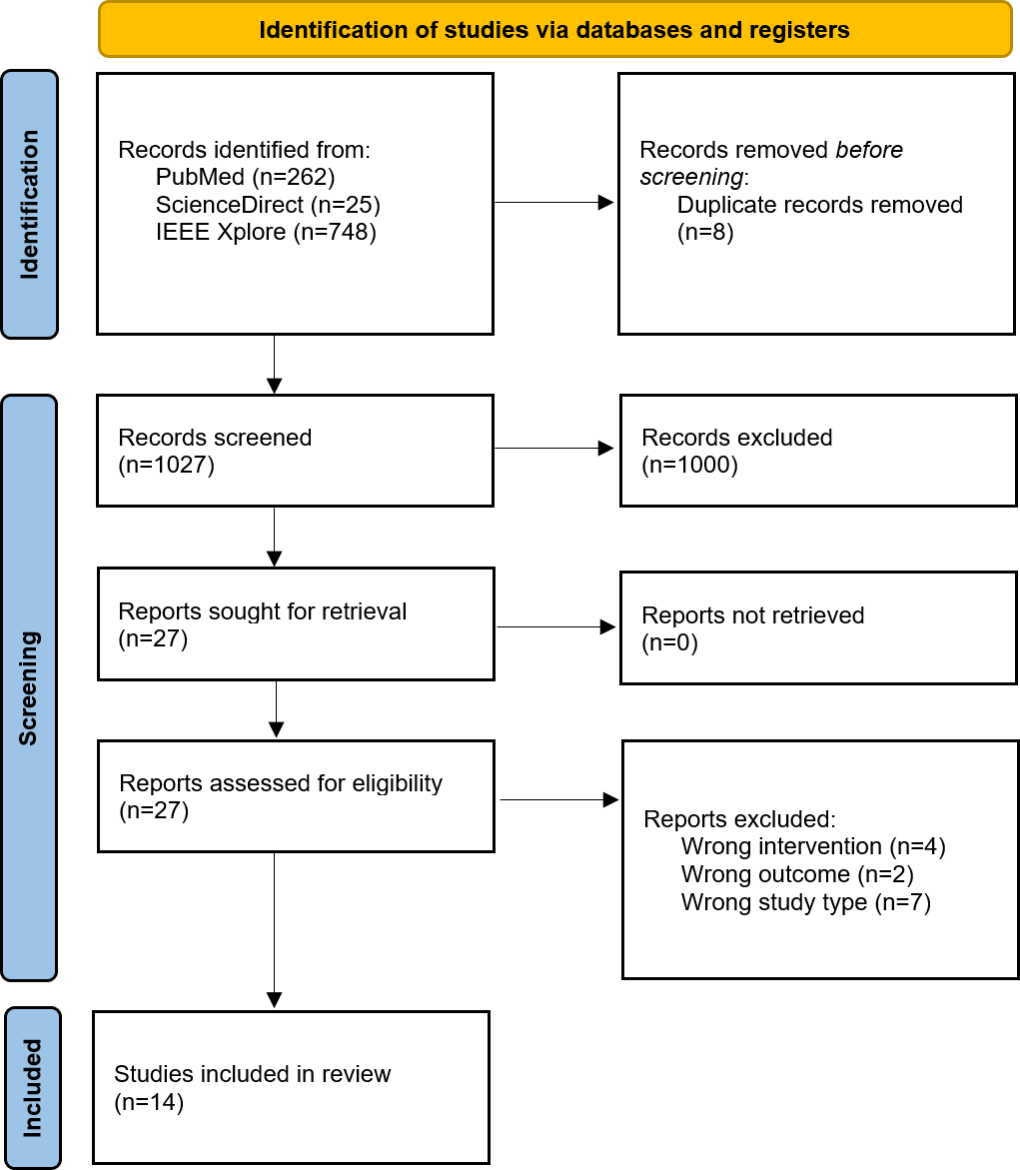
Flowchart showing the selection process.

The search in the electronic databases retrieved a total of 1035 articles, of which 8 were duplicates. Finally, the titles and abstracts of 1027 studies were screened. Based on the inclusion criteria, 27 studies were eligible for full-text review; of these, 14 studies fulfilled the criteria and were included in the scoping review. Studies excluded during the full-text analysis are listed in Multimedia Appendix 2, where the title of the paper and the reason for exclusion are indicated. Figure 1 shows the flowchart of the selection process.

Conference abstracts represented a relevant proportion of the retrieved evidence (7/15 studies), no study allowed comparison between techniques. The main fields of application were the health sector and AI applications (see Table 1).

The method adopted for design was described in 8/14 studies. In particular, HCD methodologies were adopted in 3 studies, personas in 2 studies, while the remaining methodologies were adopted in individual studies.

In 10/14 studies, a model was adopted, while in 3 studies this was not applied. Information was not available for a single study.

Design principles and properties were not described in 2 studies, in the remaining studies trustworthiness and explainability were the most frequently adopted principles (see Table 2).

The technology and type of AI were not described in 3 studies, while the remaining studies adopted very heterogeneous technologies.

Table 3 shows the characteristics of each study in terms of scope, presence of a model, design principles used, and technology.

**Table 1.** Different applications of human-centered artificial intelligence (HCAI) for design.

Field of application	Studies, n
Health	6
AI^a^ application	5
Privacy	1
General	1
Education	1
Security	1
Safety	1

a AI: artificial intelligence.

**Table 2.** Design principles and properties.

Design principles and properties	Studies, n
Trustworthiness	6
Explainability	4
Transparency	3
User needs	3
Privacy	3
Fairness	3
Safety	2
Acceptability	2
Reliability	1
Understandability	1
Protection	1
Model confidence	1
Failure	1
Errors	1
Developer needs	1
Data needs	1
Model needs	1
Interpretability	1
Accountability	1
Decision making authority	1
Emotion	1

**Table 3.** Descriptive analysis of the included studies.

Study	Type of publication	Field of application	UCD^a^ or design methods	Yes or no (model)	Yes or no (design principles or properties)	Technology or type of AI^b^
He et al, 2022 [8]	Article	Safety, security, and health	—^c^	Yes (acceptance model of TRAS^d^)	Yes (trustworthiness)	SAR^e^
Tyagi et al, 2023 [9]	Conference abstract	Education	Value-sensitive design	Yes (enhanced HCAI^f^ framework)	No	AIED^g^ application (intelligent tutoring system; exploratory learning environments, dialogue-based tutoring system, automatic writing assessment)
Holzinger et al, 2022 [10]	Conference abstract	Health	Personas for AI (explainability)	Yes (for the design and development of the personas AI)	Yes (emotion, decision-making authority and explainability as well as ethical issues are added in HAII^h^)	—
Usmani et al, 2023 [11]	Conference abstract	General	—	Yes (user empowerment, ethical consideration, and human AI collaboration)	Yes (fairness, transparency, accountability, and privacy protection)	—
Fagbola and Thakur, 2019 [12]	Conference abstract	Development of AI systems	—	No	Yes (interpretability, explainability, fairness, transparency, and safety)	Toolkit for developing AI system (FairML, Aequitas, FairTest, IBM AIFairness 360, Mash, Concept Activation Vectors, LIME, DeepLIFT, and Themis)
Beltrão et al, 2022 [13]	Conference abstract	AI-mind	Yes (HCD^i^ methods: personas, scenarios, and journey maps)	No	No	AI-based decision-support system for clinical decision
Correia et al, 2021 [14]	Conference abstract	Human-AI integration	—	Yes (acceptance model and use of technology [UTAUT^j^ and TAM^k^])	Yes (trustworthiness and acceptability)	—
Elahi et al, 2021 [15]	Article	Privacy and health	HCD methods	Yes (design shared responsibility privacy model)	Yes (privacy protection)	AAL system (ambient assisted living)
Ahmad et al, 2023 [16]	Article	Development of AI system using HCD.	Human-centered methods	Yes (human-centered AI-based software [RE4HCAI])	Yes (requirements include user needs, model needs, data needs, explainability, and trust, as well as error handling and failure mitigation)	Model applied in a system of VR (virtual reality)
Bingley et al, 2023 [6]	Article	HCAI	Qualitative survey for HCAI	—	Yes (AI developers and user’s needs [functionality, social impact, understandability, ethic, privacy, security])	—
Ventura et al, 2023 [17]	Article	Health	—	No	Yes (user needs, acceptance of technology, and perceived truthfulness)	MAIA^l^ technology
Soliman et al, 2023 [18]	Article	Health	Yes (stakeholder input, low-fidelity sketches and high-fidelity prototype for usability test)	Yes (model performance and explainability)	Yes (model confidence, trustworthiness, and explainability)	Clinical decision support application to predict heart failure patient risk of readmission
Chen et al, 2023 [19]	Article	Health care	—	Yes (for mitigating biases in AI life cycle)	Yes (Ethical, privacy protection, fairness, understandable, and transparent)	—
Kim et al, 2022 [20]	Article	Health (home care for older adults)	Yes (focus group interviews and scenarios)	Yes (human-AI collaboration and user satisfaction)	Yes (reliable, safe, and trustworthy)	DORI - older adult guided and caregiver-monitored robot

a UCD: user-centered design.

b AI: artificial intelligence.

c Not available.

d TRAS: trustworthy robots and autonomous system.

e SAR: socially assistive robot.

f HCAI: human-centered artificial intelligence.

g AIED: artificial intelligence in education.

h HAII: human–artificial intelligence interaction.

i HCD: human-centered design.

j UTAUT: Unified Theory of Acceptance and Use of Technology.

k TAM: technology acceptance model.

l MAIA: multifunctional adaptive and interactive artificial intelligent system.

The study by He et al [8] explores the critical role of trustworthiness in the acceptance of AI systems within the domains of safety, security, and health. By employing the Trustworthiness and Reliability Assessment System (TRAS) model, the research demonstrates that user acceptance is significantly influenced by the perceived trustworthiness and reliability. Socially assistive robots are used as a case study, illustrating how trust can be enhanced in AI systems within these critical fields.

Tyagi et al [9] investigates the application of AI in education through value-sensitive design methods. The study presents an enhanced HCAI framework and explores various AI applications such as intelligent tutoring systems and dialogue-based tutoring systems. The findings underscore the importance of aligning AI systems with educational values and addressing user needs to improve acceptance.

Kim et al [20], Chen et al [19], Soliman et al [18], Ventura et al [17], and Holzinger et al [10] focus on health care applications with distinct approaches. Kim et al [20] examines home care for older adults, highlighting human-AI collaboration and user satisfaction as critical design principles. Chen et al [19] address biases in the AI lifecycle within health care, emphasizing ethical principles, privacy protection, fairness, and transparency. Soliman et al [18] investigates clinical decision support systems for predicting heart failure patient readmission risks, emphasizing model performance, explainability, and trustworthiness. Ventura et al [17] explores health applications, emphasizing user needs and technology acceptance, particularly the perceived trustworthiness of AI systems. Holzinger et al [10] uses personas in the health sector to enhance AI explainability, integrating emotional and ethical considerations to improve user understanding and trust in AI technologies. These studies collectively contribute to advancing AI applications in health care by addressing various challenges and enhancing user confidence and acceptance.

Usmani et al [11] addresses general AI applications with an emphasis on user empowerment and ethical considerations. The research highlights the importance of human-AI collaboration and identifies design principles such as fairness, transparency, accountability, and privacy protection. These principles are crucial for developing trustworthy and ethical AI systems.

Fagbola and Thakur [12] and Ahmad et al [16] discuss the development of AI systems from complementary perspectives. Fagbola’s study emphasizes interpretability, explainability, fairness, transparency, and safety as crucial design principles, introducing tools like FairML and IBM AIFairness 360. Ahmad’s research focuses on human-centered methods, presenting RE4HCAI, a software that addresses user needs, model requirements, data considerations, explainability, and trust. These approaches collectively aim to foster the development of fair, transparent, and user-centric AI systems, enhancing both usability and trust in AI applications, including virtual reality environments.

Beltrão et al [13] explores the use of human-centered design methods such as personas, scenarios, and journey maps in developing AI-based decision-support systems for clinical settings. The study highlights the importance of aligning AI systems with clinical user needs to improve decision-making processes.

Correia et al [14] delves into human-AI integration, focusing on acceptance models and technology use. The study uses models like Unified Theory of Acceptance and Use of Technology (UTAUT) and technology acceptance model to evaluate user acceptance, emphasizing trustworthiness and acceptability. These insights are vital for designing AI systems that users are more likely to adopt.

Elahi et al [15] examines privacy and health applications, using human-centered design methods. The study introduces a shared responsibility privacy model, underscoring privacy protection as a key design principle. This research highlights the importance of safeguarding user privacy in health-related AI systems.

Bingley et al [6] conducts a qualitative survey on HCAI. The study emphasizes the needs of AI developers and users, addressing aspects such as functionality, social impact, understandability, ethics, privacy, and security. These insights are crucial for developing AI systems that meet diverse stakeholder requirements.

**Figure 2.**
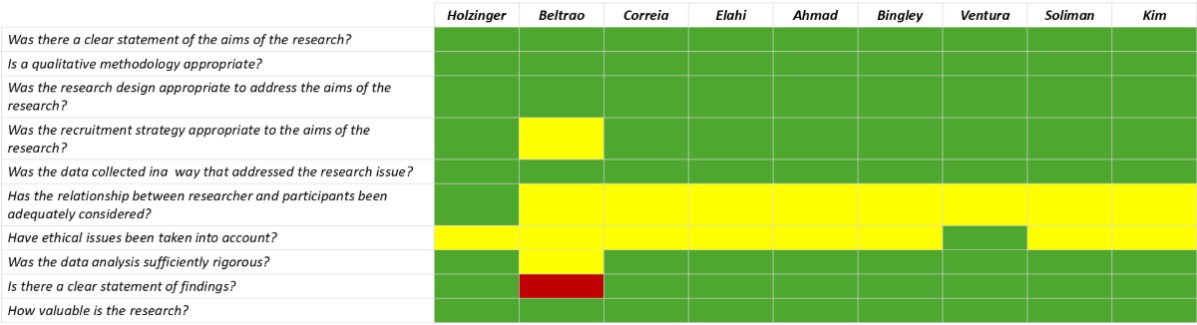
Methodological quality of the included studies presenting qualitative research, assessed with the Critical Appraisal Skill Programme (CASP) checklist for qualitative studies. Correspondence between the color and the items in the checklist: green=yes; red=no; yellow=can’t tell [6,10,13-18,20].

The methodological quality of the included studies was overall adequate, with an unclear quality of the reporting of the relationship between the researcher and participants and ethical aspects in qualitative research, while the aspect of the literature research was not explicitly addressed in most studies with a narrative design (see Figures2  3).

**Figure 3.**
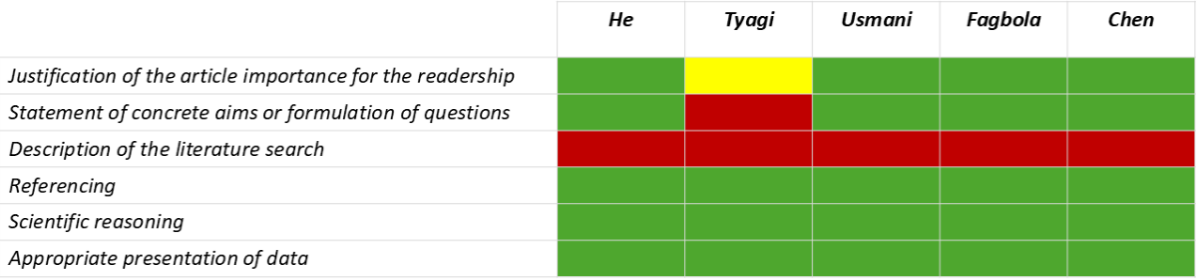
Methodological quality of the included studies presenting narrative results, assessed with the Scale for the Assessment of Narrative Review Articles (SANRA) checklist. Correspondence between the color and the items in the checklist: green=2 points; red=0 points; yellow=1 point [8,9,11,12,19].

## Discussion

### HCAI and Its Challenges in Practice

HCAI focuses on human experiences, satisfaction, and the needs, with the goal of “amplifying, enhancing, and improving human performance in ways that make systems reliable, safe, and trustworthy.” This is pivotal to “support human self-efficacy, encourage creativity, clarify responsibility, and facilitate social participation” [21].

The HCAI seeks to shift the focus in AI development, from technologies to people. However, it is unclear whether existing HCAI principles and practices adequately achieve this goal.

The results of our review indicate that although many studies address the issue of trust and acceptability in HCAI systems, few adopt systematic strategies to mitigate demographic biases and biases in health data. However, in the absence of a standardized methodology to control bias, there is a need to develop more structured guidelines to ensure trustworthiness in AI systems applied to health. In order to formalize these developments, several guidelines have been proposed by governments, organizations, and researchers to translate the ideals of HCAI into practice [22]. For example, the European Union lists 7 key requirements that AI systems should meet to be trustworthy, including transparency, accountability, and promotion of social and environmental well-being [23].

Unfortunately, despite this proliferation of guidelines, IAE ideals have proven difficult to implement [21,24,25]. At this point, Shneiderman [26] argued that although ethical guidelines are a step in the right direction, they are often too vague to be useful to software engineers. Similarly, Mittelstadt [27] criticized AI ethics for having vague principles and lofty value statements that lack the detail and precision needed to make specific recommendations for improving practice.

In line with the literature in the field, our review highlighted the need for a deeper analysis of the design principles promoted by HCAI to understand how they can be put into practice from the outset and how they differ from those proposed by HCD in terms of their impact on end consumers.

In general, it can be said that there is a need to raise the awareness of researchers and developers on the special issues offered by HCAI: the review showed that there is still a limited number of applications for AI design and solutions already available developed in accordance with HCAI principles, mainly in the field of social assistive robotics. This can partly be explained by the innovative nature of the proposed concept, but also by the partial overlap with the more general definition of human-centered design and user-centered design, highlighting the need for a clear definition and standardization of the framework.

From the analysis of recent literature [28], the explainability represents one feature deeply investigated by developers and researchers in the field of AI applications. In the health care sector, in particular, it is possible to notice a relevant focus of attention given to this principle especially from the stakeholders’ point of view. In fact, they are in charge of suggesting the use of AI systems to the final users and/or rely on AI decision support systems for complex diagnosis, for example. This evidence highlights the crucial need for transparency and understandability as the basis of explainability of AI, in particular in sensitive contexts as the healthcare sectors, in which erroneous or correct decisions have a significant impact on the well-being of older patients. At the same time, the concept of explainability overcame the mere role of technical prerequisite and became the essential driver to favor the systems’ trustworthiness.

On the end users side, in fact, it seems that the value that mostly drives and supports the uptake of AI solutions is represented by the trustworthiness, as also supported by our findings.

Our review, in fact, showed that there is the need to focus more on the concept of trustworthiness when designing a system. Even if all the articles reviewed are in accordance with observing that a user needs to trust an AI-based system in order to use it, however, they differ in the approaches to reach this outcome.

This concept is in fact multifaceted and not easily defined or achieved, as dependent from intrinsic and extrinsic characteristics of the users and of the AI technology. Several theories on technology acceptance have underlined the role of trust especially in the field of social robotics, giving AI applications a primary role in the study of the topic of trust [29,30].

Embodiment, social presence, capacity to interact following social norms and proximity, and advanced dialog features are in fact key ingredients of social assistive robots, which stimulate reciprocity and trust in the relationship between human and robot [29,30].

In addition to explainability and trustworthiness, the main concepts that are taken into consideration in the papers reviewed are transparency, privacy, and safety, which contribute to making the system more trustworthy and acceptable for the user.

The way to better achieve these aspects is still an open challenge, but this review demonstrates the importance of involving real users and stakeholders from the early conceptualization and development stage, through the entire lifecycle and testing phases of a system. This becomes a crucial aspect, especially in the development of applications to support the decision-making by health professionals in clinical fields.

Furthermore, by incorporating user feedback into the design process, users can have a sense of ownership and control over AI systems, potentially promoting trust, acceptance and better adherence, even if in most cases they have had no previous experience with AI solutions.

In addition to the need for specific HCAI methods and techniques for the design and development of new AI systems, the articles examined either did not describe the methodologies used in the design process or used the same methods as those proposed by the user-centered design approach, such as focus groups, personification, scenarios, interviews, and others, as they are independent of the type of technology while maintaining the focus on users as a pillar.

### Role of AI in Health Care

AI holds significant potential across various health care domains. AI-driven tools, particularly those based on machine learning algorithms, enhance early disease detection through advanced medical imaging analysis, such as radiography, CT, and MRI. Soliman et al [18] demonstrated the efficacy of AI in clinical decision support systems for predicting hospital readmission risk in heart failure patients, emphasizing model performance and explainability. Similarly, Chen et al [19] highlighted the use of AI to mitigate biases in healthcare applications, with a focus on ethical principles, privacy protection, and transparency. In addition, AI contributes to personalized medicine by analyzing large datasets to predict treatment responses and optimize drug dosages. Beltrão et al [13] explored AI-based clinical decision support systems, emphasizing human-centered methodologies, such as personas and journey maps [13]. Soliman et al [18] further demonstrated AI’s role in improving clinical prediction models for readmission risk.

AI also enhances patient care through virtual agents and remote monitoring systems. Kim et al [20] investigated the use of assistive robots (DORI) for home care in older adults, highlighting human–AI collaboration and patient satisfaction. Elahi et al [15] introduced a shared-privacy model for ambient assisted living applications, emphasizing privacy protection as a key design principle. Furthermore, AI-powered medical imaging applications automate routine tasks and reduce radiologists’ cognitive load. Chen et al [19] underscored AI’s role in reducing biases in image analysis. Holzinger et al [10] applied the concept of personas to improve explainability in AI systems for health care, integrating emotional and ethical dimensions to foster user trust. Despite these advancements, several challenges remain overlooked. A critical issue is the “black box” problem, referring to the opacity of AI decision-making processes, which prevents users from understanding how outcomes are generated. Although explainability and transparency have been discussed [10-12], the specific concept of the “black box”—which highlights the difficulty in interpreting model behavior—has not been directly addressed. Usmani et al [11] stressed the importance of fairness, transparency, accountability, and privacy protection in developing ethical and reliable AI systems. Fagbola et al [12] discussed tools such as FairML and IBM AI Fairness 360 to ensure algorithm interpretability and transparency. In addition, integrating AI into clinical workflows poses a challenge. Ventura et al [17] explored co-design processes with poststroke patients and caregivers, demonstrating that user involvement enhances AI solution acceptance and facilitates integration into care pathways. Correia et al [14] emphasized the importance of technological acceptance, using models like the UTAUT and technology acceptance model to assess AI’s reliability and acceptability in health care environments.

### Ethical Concerns in AI for Health Care

In addition to the technical and practical challenges, the integration of AI in health care also raises critical ethical concerns, particularly in sensitive contexts. AI decision-making in health care can have significant implications for vulnerable populations, such as those with serious or chronic conditions, the elderly, or minors. These decisions need to be made with the utmost care to avoid potential biases and discrimination. To address these concerns, AI systems must be designed with ethical safeguards that ensure fairness, transparency, and accountability, especially in critical decision-making processes.

Furthermore, AI’s role in processing sensitive patient data underscores the importance of safeguarding privacy and ensuring data security. As AI systems handle increasingly personal and confidential information, adherence to privacy laws, such as the General Data Protection Regulation, becomes essential. Health care providers must ensure that AI technologies respect patient confidentiality while maintaining trust in the system.

Another ethical challenge involves the issue of informed consent. As AI becomes more integrated into clinical decision-making, patients must be fully informed about how AI systems are used in their care. This includes understanding the role of AI in diagnostics, treatment planning, and decision support, as well as the extent to which AI influences clinical outcomes. Clear and transparent consent protocols should be developed to ensure that patients have a clear understanding of their rights and the implications of AI technologies on their care.

### Limitations and Future Directions for HCAI

Although this review provides important insights into HCAI, several limitations must be recognized. First, the number of studies focusing on HCAI remains limited and many of these overlap with human-computer interaction. This overlap hinders the differentiation between the two fields and underlines the need for further research to establish the differences between the two fields. A review of this work will be necessary in the coming years to follow the evolution of HCAI and assess the progress that will be made in establishing unique methodologies and contributions.

Another limitation is the need for a more systematic approach to define the design principles underlying HCAI. While co-design and participatory design are widely used, there is no clear guidance on how to integrate the specific principles of HCAI within these processes. Future studies should aim to develop frameworks that not only focus on technical aspects, but also address ethical, social, and contextual considerations.

Trustworthiness, explainability, and reliability remain the most critical issues for the adoption of AI-driven systems, particularly in health care. The ability to provide clear and complete explanations of AI-generated results is crucial to promote user trust. Future research should analyze practitioners’ perceptions of explainability, exploring what factors enhance or hinder trust in AI systems.

Furthermore, these emerging tools represent an opportunity to improve the participatory design process by capturing both user needs and expectations. Research is needed to understand how these tools can complement traditional HCD methods and contribute to a more user-centered AI development.

### Conclusions

HCAI emphasizes designing AI systems that prioritize human needs, satisfaction, and trustworthiness, but current principles and guidelines are often vague and difficult to implement. The review highlights the importance of involving users early in the development process to enhance trust, especially in fields like health care, but notes that there is a lack of standardized HCAI methodologies and limited practical applications adhering to these principles.

From our perspective, the future of HCAI will depend on its ability to establish itself as a structured and distinct research field. To achieve this, the development of standardized methodologies and principles tailored specifically to HCAI is essential. Rather than relying solely on existing human-centered approaches, the field should work toward codifying new design principles that account for the unique challenges posed by AI systems.

We anticipate that interdisciplinary collaboration will play a crucial role in advancing HCAI. Insights from psychology, cognitive science, and human factors engineering should be incorporated to refine HCAI methodologies, ensuring that AI systems align with human values and cognitive capabilities. Furthermore, integrating AI-based tools for user need assessment could significantly enhance the participatory design process, providing more precise and scalable insights into user expectations.

While AI in health care holds significant potential, there are critical gaps that require further investigation. Our review highlights the need for a deeper understanding of how biases in training datasets could impact equitable health care outcomes, and how improving transparency in AI decision-making is crucial for building trust. Privacy and data security concerns also need ongoing attention, particularly as AI systems increasingly handle sensitive patient information.

To bridge these gaps, future research should prioritize the integration of AI into clinical workflows, addressing potential challenges to adoption and ensuring that AI solutions are practical, ethical, and aligned with the needs of both health care providers and patients. Moving forward, interdisciplinary approaches that combine technical advancements with human-centered design will be essential for creating AI systems that are not only efficient but also ethically sound and trustworthy

## Supplementary material

10.2196/67350 Multimedia Appendix 1 Search strategies.

10.2196/67350 Multimedia Appendix 2 Studies excluded during the full text analysis indicating exclusionion motivation.

10.2196/67350 Checklist 1 Preferred Reporting Items for Systematic Reviews and Meta-Analyses Extension for Scoping Reviews (Prisma-ScR) checklist.

## Abbreviations

AI: artificial intelligence
CASP: Critical Appraisal Skills Programme
HCAI: human-centered artificial intelligence
HCD: human-centered design
OSF: Open Science Framework
PICOS: Patient, Intervention, Comparator, Outcome, and Study type
PRISMA-ScR: Preferred Reporting Items for Systematic Reviews and Meta-Analyses Extension for Scoping Reviews
SANRA: Scale for the Assessment of Narrative Review Articles
TRAS: Trustworthiness and Reliability Assessment System
UCD: user-centered design
UTAUT: Unified Theory of Acceptance and Use of Technology
