# Supplementary material for: Framing the Human-Centered Artificial Intelligence Concepts and Methods: Scoping Review
Source: JMIR Hum Factors. 2025 May 28;12:e67350. doi: 10.2196/67350 (PMC12136509; doi:10.2196/67350)
Supplement: Multimedia Appendix 1 [file humanfactors-v12-e67350-s001.docx]

| ***PubMed*** | #1 Participatory design for AI  #2 Human-centered AI for Health  #3 Human-centred AI for Health  #4 OR 1-2  #5 Human-centered Artificial Intelligence  #6 Human-centred Artificial Intelligence  #7 OR 5-6  #8 1 OR 4 OR 7  Limited to: Humans, English, 01/01/2018-27/10/2023 |
| --- | --- |
| ***ScienceDirect*** | #1 Human-centred Artificial Intelligence  #2 Human-centered Artificial Intelligence  #3 OR 1-2  #4 Human-centred AI for Health  #5 Human-centered AI for Health  #6 OR 4-5  #7 Participatory design for AI  #8 3 OR 6 OR 7  Limited to: English, 01/01/2018-27/10/2023 |
| ***IEEE Xplore*** | #1 Human-centred Artificial Intelligence  #2 Human-centered artificial intelligence  #3 Human-centred AI for Health  #4 Human-centered AI for Health  #5 OR 1-4  #6 Participatory design for AI  #7 5 OR 6  Limits: 01/01/2018-27/10/2023 |

**Supplementary table 1:** Search strategies.
