# Supplementary material for: Framing the Human-Centered Artificial Intelligence Concepts and Methods: Scoping Review
Source: JMIR Hum Factors. 2025 May 28;12:e67350. doi: 10.2196/67350 (PMC12136509; doi:10.2196/67350)
Supplement: Multimedia Appendix 2 [file humanfactors-v12-e67350-s002.docx]

**Supplementary material 2:** Studies excluded during the full text analysis indicating exclusionion motivation.

| **Ground for Exclusion** | **Exclusion motivation based on PICO criteria** |
| --- | --- |
| Description and presentation of virtual-environment simulation tool, no HCAI approaches | Intervention not HCAI for design |
| Review of guidelines of User Experience and User Interaction Design in some AI systems | Narrative review |
| Analysis of human-weel being principles, and review of ten AI ethics instruments, no valutation of HCAI approaches | Narrative review and intervention not HCAI for design |
| Narrative review about general principles of HCAI. | Narrative review |
| General comment and introduction of articles | Editorial |
| Evaluation of various AI models and algorithms in current Ambient Assisted Living technologies. | Narrative review |
| Presentation of a visual recognition technology based on artificial intelligence | Intervention not HCAI for design |
| Identification of skills needed during  the analysis process through Cognitive Task Analysis (CTA) interviews, with a perspective of future implementation of a support system based on artificial intelligence. | Different outcomes from those researched |
| Application of software engineering in a project management support system using AI methods. | Intervention not HCAI for design |
| Presentation of the current and future status of ethical and reliability issues related to human-centred AI. | Editorial |
| Review resuming the state of the art of work combining AI and IoT in the elderly population. | Narrative review |
| Presentation of a reinforcement learning system and evaluation of ethical causality in AI design. | Different outcomes from those researched |
| Introduction of an ethical technology assessment system and application of use cases in the technology verification and validation phase. | Intervention not HCAI for design |
